# Molecular Mechanisms Underlying Hull-Caryopsis Adhesion/Separation Revealed by Comparative Transcriptomic Analysis of Covered/Naked Barley (*Hordeum vulgare* L.)

**DOI:** 10.3390/ijms160614181

**Published:** 2015-06-23

**Authors:** Ruijun Duan, Huiyan Xiong, Aidong Wang, Guoxiong Chen

**Affiliations:** 1 Key Laboratory of Cold and Arid Regions Stress Physiology and Ecology in Gansu Province, Cold and Arid Regions Environmental and Engineering Institute, Chinese Academy of Sciences, Lanzhou 730000, China; E-Mails: ruijunduan@163.com (R.D.); cabin.wang@gmail.com (A.W.); 2 National Key Laboratory Breeding Base for Innovation and Utilization of Plateau Crop Germplasm, Qinghai University, Xining 810016, China; 3 College of Agriculture and Animal Husbandry, Qinghai University, Xining 810016, China; E-Mail: huiyanxqh@163.com

**Keywords:** barley, transcriptome, RNA-seq, covered/naked caryopsis, plant cuticle

## Abstract

The covered/naked caryopsis trait of barley is an important agronomic trait because it is directly linked to dietary use. The formation of covered/naked caryopsis is controlled by an NUD transcription factor, which is involved in pericarp cuticle development. However, the molecular mechanism underlying this trait remains so far largely unknown. In this study, comparative transcriptomes of grains three weeks after anthesis of Tibetan Hulless barley landrace Dulihuang and covered barley Morex were analyzed using RNA-seq technique. A total of 4031 differentially expressed genes (DEGs) were identified. The *Nud* gene was overexpressed in Morex, with trace expression in Dulihuang. Among seventeen cuticle related DEGs, sixteen were down regulated and one up regulated in Morex. These results suggest that the *Nud* gene in covered caryopsis might down regulate cuticle related genes, which may cause a permeable cuticle over pericarp, leading to a hull-caryopsis organ fusion. A functional cuticle covering the pericarp of naked caryopsis might be the result of deletion or low expression level of the *Nud* gene. The functional cuticle defines a perfect boundary to separate the caryopsis from the hull in naked barley.

## 1. Introduction

In the long domestication history of barley, many interesting traits such as non-brittle rachis, six-rowed spike and naked caryopsis appeared subsequently [1]. Among these domestication traits, the covered (hulled) or naked (hulless) caryopsis character of barley is an important agronomic trait for its linking to use purposes directly. Most cultivars have covered caryopses as animal feed and for brewing in which the hull (outer lemma and inner palea) and the pericarp epidermis are adhered firmly at maturity. However, a few cultivars are of a free-threshing varieties as human food in which the hull is departed from caryopsis at maturity easily [2]. Tibetan naked barley, is an important conventional zanba food crop which is widely cultivated and has abundant germplasm resource in the Qinghai-Tibet Plateau, China [3,4].

Originally Harlan and Hulton reported that a sticky adhesive substance appears 10 days after flowering on the caryopsis surface in hulled barley [5]. Transmission electron microscopy showed that this substance is secreted from the pericarp epidermis two days after flowering and its thickness increases during grain development [6]. Taketa and his colleagues reported that this substance is cuticular lipid, and the presence or absence of the lipid layer on the pericarp epidermis is critical for distinguishing covered and naked barley [7,8,9]. A single recessive gene, *nud*, located on chromosome 7HL [10], was found to control the naked caryopsis character, suggesting that easy separation of the hull results from a *nud* mutation that damaged gene function. Through positional cloning, the *Nud* (allele for covered caryopsis) gene is found to encode an ethylene response factor (ERF) family transcription factor and shows high similarity to the Arabidopsis WIN1/SHN1 transcription factor gene, whose deduced function is control of a lipid biosynthesis pathway [9]. However, the molecular mechanism underlying this hulled/naked character remains largely unknown.

Cuticular waxes and cutin form the cuticle, a hydrophobic layer covering the aerial surfaces of land plants and acting as a protective barrier against environmental stresses. So far, about 55 genes involved in the formation of the cuticle have been isolated, which are divided into three categories: regulatory genes, synthesis genes, and transporter genes [11,12,13,14]. In barley, >1580 *eceriferum* (*cer*) mutants with different degrees of reduced wax in different parts, are classified into 79 loci [15,16]. Three barley *cer* mutants (*cer-yl*, *cer-ym* and *cer-zv*) show weak hull-caryopsis adhesion [17], suggesting a link between epidermal cuticle lipids and hull-caryopsis adhesion [9].

In order to further understand the molecular mechanism of covered/naked caryopsis trait, comparative transcriptome profile of developing caryopses of Tibetan Hulless barley landrace “Dulihuang” and covered barley cultivar “Morex” were analyzed by RNA-seq technique. We found that the NUD transcription factor might down regulate cutcle biosynthesis genes in covered barley, and that deletion or low expression level of the *Nud* gene may result in the normal expression of cuticle related genes in naked barley.

## 2. Results

### 2.1. The RNA-Seq Data Were Highly Reproducible

To compare transcriptomes of covered caryopsis of hulled barley cultivar Morex and naked caryopsis of Tibetan Hulless barley landraces Dulihuang at three weeks after anthesis, cDNA libraries were prepared from caryopsis (including hulls) and subjected to RNA-Seq analysis on the Illumina HiSeq 2000 platform. Through the further barley genome deciphering [18], the RNA-Seq technology can adopt the method of a reference genome, which would make the results more reliable. In total, 136.3 million short reads were generated from the two genotypes, with 96.7 million high-quality 90-bp clean reads selected for further analysis. After removing the low-quality sequences and trimming the adapter sequences, a total of 48 million clean reads were generated for each of the respective libraries (Table 1).

**Table 1:** Read number and statistics based on the RNA-Seq data in the two libraries of barley varieties.

Map to Genome	Dulihuang Reads Number	Morex Reads Number
Total clean Reads	48,428,404	48,350,614
Total Base Pairs	4,358,556,360	4,351,555,260
Total Mapped Reads	26,026,979	24,135,038
Perfect match	12,807,926	15,195,341
≤5 bp mismatch	13,219,053	8,939,697
Unique match	12,908,475	13,718,448
Multi-position match	13,118,504	10,416,590
Total Unmapped Reads	22,401,425	24,215,576
Genome map Rate	53.74%	49.92%
Gene map Rate	21.96%	17.52%
Expressed Genes	20,420	20,372

Clean reads were mapped to the barley (*Hordeum vulgare* L.) genome ([http://www.ncbi.nlm.nih.gov/] [ftp://ftpmips.helmholtz–muenchen.de/plants/barley]) by using SOAPaligner/soap2, with no more than five base mismatches allowed in the alignment. Of the total clean reads obtained from Dulihuang and Morex, 26.45% and 31.43% reads were perfect matches, 27.3% and 18.49%, respectively, had 1–5 bp mismatches to the reference genome, while 46.26% and 50.08% reads respectively were unmapped to the reference genome (Table 1). Of the mapped reads, 26.65% matched to unique positions, and 27.09% matched to multiple genome locations in Dulihuang, while 28.37% matched to unique and 21.54% matched to multiple genome locations in Morex.

**Figure 1:**
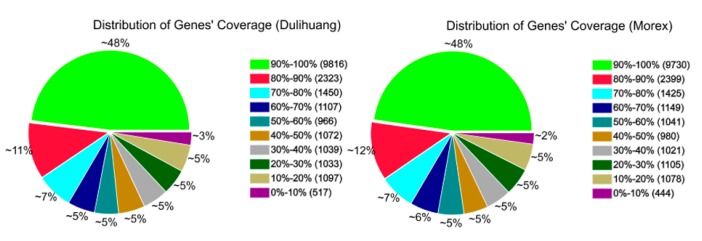
Gene coverage of sample Morex and Dulihuang reads.

Gene coverage, the ratio of the base number in a gene, covered by unique mapping reads to the total number of bases of that gene, can reflect the quality of sequencing. In this experiment, the gene coverage over the Morex and Dulihuang RNA-seq libraries was highly reproducible and quite uniform (Figure 1), indicating that this RNA sequencing was of a high quality.

By comparison with the barley genome, all 97 million clean reads were assembled into 20,372 genes for Morex and 20,420 genes for Dulihuang, providing massive data for further analysis (Table 1).

### 2.2. Significantly Differentially Expressed Genes (DEGs) Were Found in the Comparison between Dulihuang and Morex

**Figure 2:**
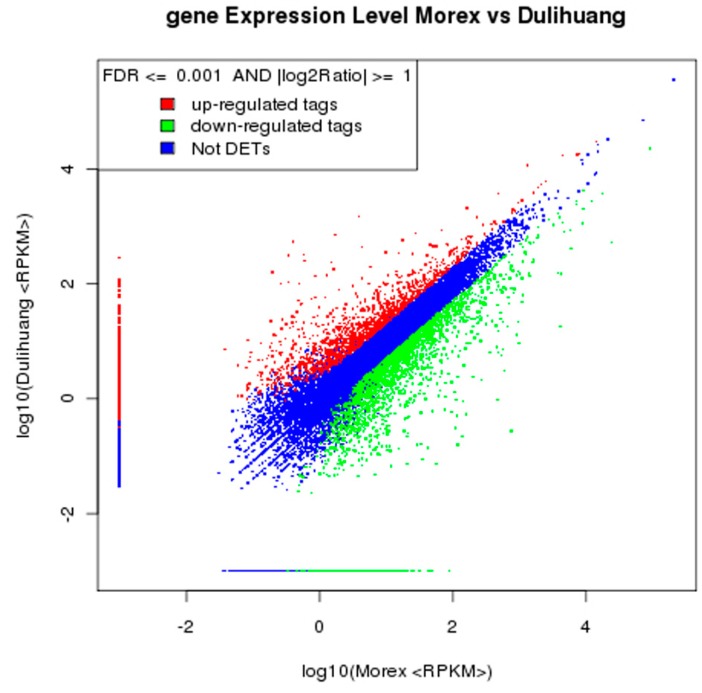
DEG analysis of Dulihuang and Morex libraries. All genes mapped to the reference sequence were examined for differences in their expression across the two libraries. *X*-axis and *Y*-axis represent the two samples log10 value of expression, red (up) and green (down) dots denote that the expression was significantly different (FDR ≤ 0.001, 2-fold difference), whilst the blue dot indicates that there was no significant difference between the expression levels in the two varieties.

Gene expression levels were calculated by using the RPKM (Reads per kb per million reads) method. Their detailed expression is shown in Table S1. We focused the analysis on the differentially expressed genes (DEGs) that had relatively high abundance and exhibited expression that was differential between the two genotypes. DEGs were defined as the fold-change value of the normalized RPKM at log2Ratio ≥1 and the false discovery rate (FDR) ≤0.001. Using ERANGE (version 4.0) (http://woldlab.caltech.edu/gitweb/), we identified 4031 DEGs in Morex-vs-Dulihuang (Table S2). Among the DEGs, 2510 genes were upregulated and 1521 genes were downregulated in Morex in comparison to Dulihuang (Figure 2).

### 2.3. Validation of DEGs

**Figure 3:**
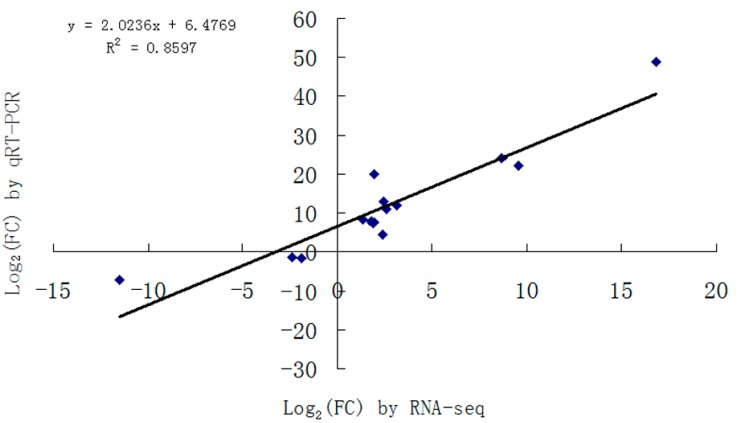
Comparison of log_2_(FC) of 15 selected transcripts using RNA-seq and qRT-PCR.

The validation of DEGs was conducted by quantitative real-time PCR (qRT-PCR). A subset of 15 DEGs in Morex and Dulihuang were randomly selected for qRT-PCR. Their primers were designed on the basis of barely sequences obtained as results from RNA-Seq; their primer sequences are listed in Table S3. We compared their expression results obtained from qRT-PCR with those generated by the RNA-Seq. The expression trends were consistent for all transcripts in both analyses, with a correlation coefficient *R*^2^ = 0.8597 (Figure 3). This consistency indicates that the DEG analysis is trustworthy, and its results are suitable for further investigation.

### 2.4. Functional Classification of DEGs

To acquire functional information of DEGs, BLAST2GO (version 2.3.5) was used for Gene Ontology (GO) functional classification. Within the biological process category, DEGs were primarily assigned to GO terms of metabolic process (1145 genes), cellular process (919 genes), and response to stimulus (322 genes). In the cellular component category, most DEGS fell into cell (1716 genes), cell part (1716 genes) and organelle (1472 genes). In the molecular function category, the major GO terms were binding (1177 genes) and catalytic activity (1329 genes) (Table S4). These major GO terms were similar to those of DEGs between two Tibetan hulless caryopses XQ754 and Nimubai [19], indicating that the majority of DEGs are not related to covered/naked caryopsis trait. The covered/naked caryopsis trait is determined by hull-caryopsis adhesion/separation [6], which is related to the development of pericarp cuticle [20]. Thus, we analyzed the DEGs associated with pericarp cuticle development.

### 2.5. Differentially Expressed Known Cuticle Genes

About 55 cuticle genes have been cloned in Arabidopsis, maize and other plants (Table S5). Their corresponding barley homologus genes were found in the RNA-seq results and 18 of them were DEGs (Table 2). The *Nud* gene encodes an ERF transcription factor that is involved in hull-caryopsis adhesion in hulled barley, while its mutant *nud* exhibits a separation between the hull and caryopsis in hulless barley [9]. Accordingly, the *Nud* gene was overexpressed in Morex with trace expression in Dulihuang, which was consistent with its role in the development of hulled barely. *CER9* gene was also overexpressed in Morex. Except for *Nud* and *CER9*, all the other cuticle genes were downregulated in Morex. About 56 percent of differentially expressed cuticle related genes were associated with increased cuticle permeability and organ fusion (Table 2). We speculate that the NUD transcription factor downregulates cuticle genes to form a disorganized pericarp cuticle that leads to the hull-caryopsis organ fusion in hulled barley.

## 3. Discussion

We carried out a transcriptome analysis of caryopses of hulled and hulless barley, Morex and Duliuang, respectively, to reveal genes that may be involved in hull-caryopsis adhesion. A total of 4031 DEGs were identified, most of which were classified into three groups, metabolic process, cellular process, and response to stimulus, in biological process category of gene ontology (GO) functional classification. The same three top groups are also found in DEGs in caryopses of two Tibetan hulless barley varieties [19], indicating that the majority of DEGs in the present study were not related to covered/naked caryopsis trait. Most of the DEGs may reflect many other caryopsis traits varied between Morex and Dulihuang. A cementing layer causes hull-caryopsis adhesion in covered caryopses [9]. This cementing layer is a thickened and loose cuticle of pericarp [6,20]. Thus, we focused on DEGs associated with cuticle development.

Expression level of *Nud* gene was much more pronounced in Morex than in Dulihuang. This result confirms that the *Nud* gene is responsible for hull-caryopsis adhesion in hulled barley [9]. NUD protein is a transcription factor related to regulation of lipid biosynthesis pathway. It may regulate expression of genes involved in the development of pericarp cuticle in hulled barley, but not in hulless barley, because the *Nud* gene is deleted in all 100 hulless barley cultivars tested [9] and the origin of hulless barley is monophyletic [7,9]. To our surprise, the *Nud* gene was found to be expressed in hulless barley cultivar Dulihuang. We investigated caryopsis RNA-seq results (NCBI Sequence Read Archive, Accession numbers: SRR1032035, SRR1032036, SRX375649 and SRX378862) of two Tibetan hulless barley cultivars [19], and found that the *Nud* gene was also expressed in one of them. The genomic sequence of the *Nud* gene in Dulihuang and the other hulless barley will be analyzed to confirm its existence in hulless barley. There might be mutations in the two *Nud* sequences or posttranslational regulation of the two NUD protein, which lead to the naked phenotype.

**Table 2:** Differentially expressed cuticle related genes.

Gene Name	Gene ID	Plant	Function	Mutant Phenotype		Barley Gene	Log2 Ratio (Morex/Dulihuang)	FDR
				Cuticle Permeability	Organ Fusion			
*Nud*	*GU108424*	*Barley*	Regulation	No	No	MLOC_59305.1	+5.13	5.78 × 10^−18^
*SHN1*	*At1g15360*	*Arabidopsis*	Regulation	Permeable	Organ fusion	MLOC_12747.2	−3.12	2.48 × 10^−10^
*ATT1*	*AT4G00360*	*Arabidopsis*	Cutin biosynthesis	Permeable	No	MLOC_71597.1	−2.53	2.84 × 10^−273^
*GPAT6*	*AT2G38110*	*Arabidopsis*	Cutin biosynthesis	Permeable	Organ fusion	MLOC_56115.1	−1.93	8.71 × 10^−51^
*GPAT8*	*AT4G00400*	*Arabidopsis*	Cutin biosynthesis	Permeable	Organ fusion	MLOC_19148.1	−4.38	1.36 × 10^−5^
*LACS2*	*AT1G49430*	*Arabidopsis*	Cutin biosynthesis	Permeable	Organ fusion	MLOC_66827.1	−2.42	1.15 × 10^−39^
*LCR*	*AT2G45970*	*Arabidopsis*	Cutin biosynthesis	Permeable	Organ fusion	MLOC_75020.2	−1.37	2.27 × 10^−7^
*HTH*	*AT1G72970*	*Arabidopsis*	Cutin biosynthesis	Permeable	Organ fusion	MLOC_9891.3	−1.78	1.89 × 10^−6^
*FDH1*	*AT2G26250*	*Arabidopsis*	Wax biosynthesis	Permeable	Organ fusion	MLOC_65700.1	−1.96	1.47 × 10^−280^
*WAX2*	*AY131334*	*Arabidopsis*	Unknown	Permeable	Organ fusion	MLOC_51499.1	−1.05	2.16 × 10^−6^
*CER9*	*At4g34100*	*Arabidopsis*	Unknown	No	No	MLOC_36840.2	+1.10	5.29 × 10^−46^
*ACC1*	*AT1G36160*	*Arabidopsis*	Wax biosynthesis	Permeable	Organ fusion	MLOC_37244.1	−1.63	5.61 × 10^−47^
*EIBI1*	*AB534898*	*Barley*	Secretion	Permeable	-	MLOC_62487.1	−1.63	5.43 × 10^−86^
*WBC11*	*At1g17840*	*Arabidopsis*	Secretion	Permeable	Organ fusion	AK355515	−1.10	4.35 × 10^−12^
*MYB96*	*At5G62470*	*Arabidopsis*	Regulation	No	No	MLOC_34885.1	−1.72	5.80 × 10^−9^
*WSD1*	*AT5G37300*	*Arabidopsis*	Wax biosynthesis	No	No	MLOC_74286.1	−1.86	7.78 × 10^−15^
*CUT1*	*AT1G68530*	*Arabidopsis*	Wax biosynthesis	No	No	MLOC_51583.2	−2.36	4.61 × 10^−34^
*FATB*	*AT1G08510*	*Arabidopsis*	Fatty acids biosynthesis	No	No	MLOC_76304.1	−1.21	0.00015

NUD might regulate one transcription factor gene (*SHN1*), six cutin biosynthesis genes (*ATT1*, *LCR*, *GPAT6*, *GPAT8*, *LACS2*, and *HTH*), four cutin related genes (*FDH1*, *WAX2*, *CER9*, and *ACC1*), and two cutin transporter genes (*Eibi1* and *WBC11*). SHN1 primarily controls cutin biosynthesis by regulating cutin related genes, and indirectly affect wax accumulation [21]. ATT1 and LCR are most likely involved in the cutin monomer biosynthesis as cytochrome P450s of the CYP86A type [22,23]. GPAT6 and GPAT8 are sn-2 acyl transferase with glycerol-3-phosphate as the acyl acceptor [24]. LACS2 is a long chain acyl-CoA synthetase required to activate free fatty acids to the acyl-CoAs [25]. HTH is an oxidoreductase that is thought to be involved in the biosynthesis of dicarboxylic acids [26]. Although FDH has been identified as a β-ketoacyl-CoA synthase based on sequence similarity [27], its mutant *fdh* shows an increase in cutin constituents [28]. The biochemical role of FDH in cutin formation has not yet been elucidated [29]. WAX2 is a protein of unknown function required for cuticular wax biosynthesis; it also may be necessary for cutin synthesis [30]. CER9 is an E3 ubiquitin ligase; loss of its function leads to coordinated alterations in cutin and wax biosynthesis and improved drought resistance of the cuticle [31]. ACC1, an acetyl coenzyme A carboxylase, plays a primary role in the biosynthesis of very-long-chain fatty acids associated with cuticular waxes; its mutant allele *acc1*/*gsd1* has less amount of wax but more cutin deposition on inflorescence stems [32]. Eibi1 is a full ABCG transporter identified in barley and WBC11 is a half ABCG transporter identified in Arabidopsis, both for cutin deposition [33,34]. Mutations (*shn1*, *gpat6*, *lacs2*, *lcr*, *hth*, *fdh1*, *wax2*, *acc1* and *wbc11*) in most of the above-mentioned genes, lead to two common phenotypes, enhanced cuticle permeability and organ fusion. NUD may down-regulates the expression of cutin related genes (*SHN1*, *GPAT6*, *GPAT8*, *LACS2*, *LCR*, *HTH*, *FDH1*, *WAX2*, *WBC11*, and *ATT1*) to make a permeable cuticle proper, which caused to the hull-caryopsis fusion in hulled barley. *CER9* is the only upregulated cutin linked gene in covered caryopses; it is a unique gene whose mutation improves hydrophobic barriers to water diffusion through cuticle membrane [31]. An upregulation of *CER9* gene expression may cause a more permeable cuticle, which has advantages for hull-caryopsis fusion in hulled barley. Taken together, these data suggest that NUD regulation of cutin biosynthesis pathway may be involved in covered caryopsis development.

The pericarp cuticle of covered caryopsis (figure 37 in [6]) is reminiscent of the cuticle proper of organ fusion mutants, such as *wax2* and *wbc11*, which is thicker with a loose and less osmiophilic structure (figure 1 in [30]; figure 7e in [34]), while the pericarp cuticle of naked caryopsis (figure 9 in [6]) is comparable to wild-type cuticle (figure 7d in [34]). Insoluble cutin is the major structural material of plant cuticle [35]. The thick and loose cutin is able to explain the increased permeability of pericarp cuticle in covered caryopses rather than in the naked one [9]. High permeable cuticle of pericarp may cause fusion of the hull and caryopsis. We may conclude that hulled barley displays hull-caryopsis fusion instead of adhesion.

A naked caryopsis, without the hull protection, has to build up a functional cuticle to seal itself to prevent water loss. A transcription factor MYB96 was upregulated in hulless Dulihuang. MYB96 improves plant drought resistance by promoting cuticular wax accumulation under drought stress; it binds directly to the promoters of wax biosynthetic genes and subsequently activating their transcription. The upregulated wax biosynthetic genes by MYB96 include *WSD1*, *FATB*, and *CUT1* [36]. These three genes were also upregulated in Dulihuang. We therefore propose that the MYB96 regulation of wax biosynthesis pathway may be involved in naked caryopsis development.

## 4. Experimental Section

### 4.1. Plant Material

For transcriptomic analyses, barley varieties Morex and Tibetan Hulless barley Landrace Dulihuang were used. Two varieties were planted in a field of Academy of Agriculture and Forestry, Qinghai University. At grain filling stage, three replicates of three-week-old caryopses (including hulls at three weeks after anthesis) from both varieties were collected for RNA extraction.

### 4.2. RNA Extraction, cDNA Library Preparation, and Sequencing

Total RNA was prepared using Trizol according to the manufacturer’s protocol (Invitrogen, Burlington, ON, Canada) with some modification. The concentration of each RNA sample was determined by NanoDrop 2000TM micro-volume spectrophotometer (Thermo Scientific, Waltham, MA, USA) and gel electrophoresis. The RNA quality was checked on a Bioanalyzer 2100 (Aligent, Santa Clara, CA, USA); RNA Integrity Number (RIN) values were greater than 8.5 for all samples.

Sequencing libraries were prepared according to the manufacturer’s instructions (Illumina, San Diego, CA, USA).

Primary sequencing data produced by Illumina HiSeqTM 2000 (Illumina, San Diego, CA, USA), called raw reads, is subjected to quality control (QC) to determine if a resequencing step is needed. The raw reads were cleaned by removing adaptor sequences, empty reads and low quality sequences.

During the quality control (QC) steps, the 2100 Bioanylzer (Agilent Technologies, Santa Clara, CA, USA) and ABI StepOnePlus Real-Time PCR System (Applied Biosystems, Foster City, CA, USA) were used in quantification and qualification of the sample library. Finally, the library is sequenced using Illumina HiSeqTM 2000.

### 4.3. Functional Annotation: Evaluation of Genes from RNA-Seq

After QC, raw reads were filtered into clean reads to be aligned to the barley reference sequences with SOAPaligner/SOAP2 [37]. The alignment data was utilized to calculate distribution of reads on reference genes and perform gene coverage analysis. After results have passed QC, downstream analysis including gene expression and gene annotation were performed. Results of gene expression include gene expression levels and differential expression analysis. Further, we perform Gene Ontology (GO) enrichment analysis.

Raw digital gene expression counts were normalized by a variation of the RPKM (Reads per kb per million reads) method, which could eliminate the influence of different gene length and sequence discrepancy on the calculation of gene expression. Therefore, the calculated gene expression could be directly used for comparing the difference of gene expression among samples. For all RPKM values of each gene, the cutoff value for determining gene transcriptional activity was determined based on a 95% confidence threshold. The Blast2GO program was used to obtain GO annotations (version 2.3.5) for the all genes [38]. Then, the software WEGO was used to perform GO functional classification of all genes to view the distribution of gene functions of the species at the macro level with the default parameters with a robust FDR correction to get an adjusted *p* value [39].

### 4.4. Validation of RNA-Seq by qRT-PCR

Total RNA from two samples (Morex and Dulihuang) was treated with DNase, and first-strand cDNA was generated using an AMV First Strand cDNA Synthesis Kit (Sangon, Shanghai, China). SYBR-based qRT-PCR reactions (SYBR Green I, ABI, Foster City, CA, USA) were performed on a LightCycler 480 system (Roche, Basel, Switzerland) using the following reaction conditions: 95 °C for 3 min followed by 40 cycles of 95 °C for 15 s and 60 °C for 40 s. All qRT-PCR reactions were performed in triplicate, and the results were analyzed with the system’s relative quantification software (ver.1.5) based on the 2^−∆∆*C*t^ method (Roche). The primers were listed in Table S3. The detection threshold cycle for each reaction was normalized against the expression level of the HvActin gene with primer sequences 5′-AAGTACAGTGTCTGGATTGGAGGG-3′ and 5′-TCGCAACTTAGAAGCACTTCCG-3′.

## 5. Conclusions

In summary, we used RNA-Seq to analyse differential expression genes (DEGs) underlining the covered/naked caryopsis trait in barley, and 4031 genes are differentially expressed between Morex/Dulihuang. Due to the majority of DEGs not being related to the covered/naked caryopsis trait, we analyzed the DEGs associated with pericarp cuticle development, which is related to hull-caryopsis adhesion/separation. NUD transcription factor regulates the cutin biosynthesis pathway, leading to a thick and loose cutin, which forms a high permeable cuticle on the pericarp of covered caryopsis. The high permeable cuticle is responsible for the hull-caryopsis fusion in hulled barley. A functional cuticle covering the pericarp of naked caryopsis is the result of deletion or low expression level of *Nud* gene and MYB96 regulation of wax biosynthesis pathway. The functional cuticle defines a perfect boundary of a pericarp to separate the caryopsis from the hull in hulless barley. The major findings in our study provide a good starting point for future functional study involved in the covered or naked caryopsis trait.

## Supplementary Materials

Supplementary materials can be found at http://www.mdpi.com/1422-0067/16/06/14181/s1.
